# The Effect of Consistency on Short-Term Memory for Scenes

**DOI:** 10.3389/fpsyg.2017.01712

**Published:** 2017-10-04

**Authors:** Mingliang Gong, Yuming Xuan, Xinwen Xu, Xiaolan Fu

**Affiliations:** ^1^State Key Laboratory of Brain and Cognitive Sciences, Institute of Psychology, Chinese Academy of Sciences, Beijing, China; ^2^Department of Psychology, Miami University, Oxford, OH, United States; ^3^Department of Psychology, University of the Chinese Academy of Sciences, Beijing, China

**Keywords:** scene consistency, short-term memory, change detection, semantic content, scene gist

## Abstract

Which is more detectable, the change of a consistent or an inconsistent object in a scene? This question has been debated for decades. We noted that the change of objects in scenes might simultaneously be accompanied with gist changes. In the present study we aimed to examine how the alteration of gist, as well as the consistency of the changed objects, modulated change detection. In Experiment 1, we manipulated the semantic content by either keeping or changing the consistency of the scene. Results showed that the changes of consistent and inconsistent scenes were equally detected. More importantly, the changes were more accurately detected when scene consistency changed than when the consistency remained unchanged, regardless of the consistency of the memory scenes. A phase-scrambled version of stimuli was adopted in Experiment 2 to decouple the possible confounding effect of low-level factors. The results of Experiment 2 demonstrated that the effect found in Experiment 1 was indeed due to the change of high-level semantic consistency rather than the change of low-level physical features. Together, the study suggests that the change of consistency plays an important role in scene short-term memory, which might be attributed to the sensitivity to the change of semantic content.

## Introduction

Objects always appear in certain contexts—a toaster is usually seen in the kitchen while a pillow is usually seen on the bed. We gradually gain experience about where a particular object often appears which becomes a part of our knowledge structure, or scene schema (Mandler and Ritchey, 1977; Henderson, 2003). The probability of an object appearing in a context is called scene consistency, with a consistent scene corresponding to a high probability while an inconsistent scene corresponding to a low probability.

Previous studies have shown that scene consistency plays an important role in the processing of scenes. In early 1970s, Biederman and colleagues conducted a series of studies showing that coherent context facilitated object identification (Biederman, 1972; Biederman et al., 1974) and visual search (Biederman et al., 1973) compared to scrambled context. The conclusion was confirmed by many later studies (for review, see Bar, 2004; Oliva and Torralba, 2007; Zimmermann et al., 2010; LaPointe and Milliken, 2016 but see Hollingworth and Henderson, 1998, 1999). Other studies directly compared the processing of objects in consistent versus inconsistent scenes and found that objects are recognized faster and more accurately when foreground objects are consistent with background contexts than when they are inconsistent (Biederman et al., 1982; Boyce et al., 1989; Davenport and Potter, 2004; Davenport, 2007; Joubert et al., 2008; Mudrik et al., 2010), a phenomenon called “scene consistency effect.” For example, Davenport and Potter (2004) adopted color pictures of scenes to examine the effect of consistency on a naming task. Scene picture were presented for only 80 ms and followed by a mask, and then participants were required to name either the object (e.g., camel) or the background (e.g., desert). The results showed that both objects and backgrounds were named more accurately when they were in consistent scenes than when they were in inconsistent scenes. ERPs studies showed that an N400-like component was evoked by inconsistent scenes, which confirmed that the differences between the processes of consistent and inconsistent scenes were indeed due to the semantic relationship between foreground object and background (Ganis and Kutas, 2003; Sitnikova et al., 2003, 2008; Mudrik et al., 2010). Together, these studies suggest that consistent scenes facilitate object recognition.

Intriguingly, most studies demonstrated that objects in consistent scenes, though having an advantage in identification, showed a disadvantage in capturing attention compared to objects in inconsistent scenes. First, it is well-established by eye movement studies that scene consistency influences attention orienting, with inconsistent scenes being fixated earlier and for a longer time than consistent scenes (Loftus and Mackworth, 1978; De Graef et al., 1990; Underwood and Foulsham, 2006; Brockmole and Henderson, 2008; Underwood et al., 2008; Spotorno et al., 2015; LaPointe and Milliken, 2016). For example, Brockmole and Henderson (2008) added new objects to real-world scenes during a fixation or during a saccade. The objects were either consistent or inconsistent with the scenes in terms of meaning. They found that inconsistent new objects were fixated sooner than consistent ones. The second line of research derives from binocular rivalry studies (Mudrik et al., 2011a,b). When two images that shared the same background but had different objects—one consistent and one inconsistent—were presented in isolation to each eye, inconsistent objects predominated in awareness longer than consistent ones in rivalry (Mudrik et al., 2011b). Similarly, objects inconsistent with scenes escaped from perceptual suppression faster than consistent ones in a continuous flash suppression paradigm (Mudrik et al., 2011a).

However, Moors et al. (2016) failed to replicate Mudrik colleagues study and did not show the inconsistent object advantage. A more recent study employed four tasks (inattention, scene description, change detection and iconic memory) to investigate this question and showed that this inconsistent object advantage only appeared in the change detection task. Since participants took a long time (11 s on average) to detect the changes, the authors proposed that this advantage was not because incosistent objects had an advantage in capturing initial attention; rather it was because attention dwelled on inconsistent objects longer once attention landed on them when the change was detected (Mack et al., 2017).

Together, though the evidence that supports the advantage of inconsistent objects in capturing initial attention is not undisputed, most studies suggest that inconsistent objects have an advantage in capturing initial attention. Therefore, previous studies seem to show a dissociation between the speed of perceptual processing and attentional orientation for consistent versus inconsistent scenes: while inconsistent objects tend to attract attention earlier than consistent ones, consistent objects are processed faster. The paradoxical results aroused our interests in investigating the effect of consistency on the memory of scenes. On the one hand, people’s everyday experience gives rise to the formation of scene schemas stored in long-term memory—the representations of co-occurrence relationship between scenes and particular objects as well as their spatial relationships (Chun, 2000), so consistent scenes are more familiar to people. In this sense, consistent scenes should facilitate visual short-term memory (VSTM; Familiarity hypothesis). On the other hand, inconsistent scenes are novel stimuli, and since novelty enhances VSTM encoding (Mayer et al., 2011), inconsistent scenes rather than consistent scenes should benefit VSTM (Novelty hypothesis). Together, both consistent and inconsistent scenes possess their advantages in being maintained in VSTM, yet how consistency influences scene VSTM is by no means resolved.

The first purpose of this study was to investigate the role of consistency in scene VSTM. Only a few studies have tapped short-term memory for scenes but showed contradictory results. In a flicker paradigm, Hollingworth and Henderson (2000) found that if an object disappeared or changed orientations, or a new object appeared, detection latency was shorter when the object was semantically inconsistent with the background than when it was consistent. They speculated that semantic features of an object modulated the retention of object representation in working memory (Hollingworth and Henderson, 2000). Subsequent studies either showed the same results (Hollingworth and Henderson, 2003; Stirk and Underwood, 2007) or the opposite (Spotorno et al., 2013). For instance, Spotorno et al. (2013) used a one-shot change detection task and showed that high consistent objects, when added to or deleted from a scene, were more accurately detected than inconsistent ones.

The advantage of consistent or inconsistent scenes can depend on the task requirements—there was a benefit for consistent scenes when identifying the changing object while a benefit for inconsistent scenes when detecting and localizing the object (LaPointe et al., 2013). This result is in accordance with the dissociation between the advantage of consistent scenes in the speed of perceptual processing and the advantage of inconsistent scenes in attentional capture. However, the benefit of inconsistent scenes in detection task showed by Spotorno et al. (2013) seems to contradict the conclusion. Furthermore, most of these studies addressed the detection of changes from an attentional or a perceptual perspective rather a memorial perspective, though apparently change detection tasks require short-term memory. Therefore, it is still an open question whether consistent or inconsistent scenes facilitate VSTM or how they modulate VSTM.

Another possible explanation for the previous results is from the perspective of gist. The gist of a scene, i.e., the semantic meaning or the category of the scene, is determined by the background and objects in the scene. It can be extracted at a very brief glimpse (Rousselet et al., 2005; Oliva and Torralba, 2006; Joubert et al., 2007; Castelhano and Henderson, 2008; Greene and Oliva, 2009). The quickly extracted gist information provides a context that guides the allocation of attention toward potential target objects within the scene (Chun, 2000; Bar, 2004; Joubert et al., 2008; Võ and Henderson, 2010; Gong et al., 2011). Therefore, gist extraction is vital to the perception of a scene. This claim is supported by empirical research showing that changes that also alter the gist of a scene are more likely to be detected compared to change that do not alter the gist, indicating that gist plays an important role in scene recognition (Sampanes et al., 2008). One of the scenes used in the study was a log immediately in the path of a man kayaking down a river. It had two changes: the log was changed to a kayak; the log was changed to a rock. It was found that participants were more readily to detect the former change compare to the latter because the gist was altered in the former change but not the latter. It is worth noting that rock and log are unmovable, which may signify potential danger when they are immediately in the path of kayaking. By contrast, the kayak is movable so no signal of danger is conveyed if a second kayak is immediately in the path of kayaking. Thus the gist was altered when the log was changed to a second kayak but not altered when the log was changed to a rock. This study suggests that the alteration of gist can modulate change detection. However, previous research has failed to take the alteration of meaning (i.e., gist) of the scene into account while they were examining the consistency effect in the detection of scene changes. In previous studies, participants were asked to detect either the addition or deletion of an object, which might also alter the gist of a scene. It is likely that the change of different objects may affect the gist to varying degrees. For instance, whereas the addition of a microwave oven in a kitchen setting does not alter the gist, the addition of a toilet does.

In the present study, we adopted a masked one-shot change detection paradigm to examine the effect of scene consistency, as well as the change of semantic content, on VSTM. The first purpose of the study was to examine the controversy of whether consistent or inconsistent scene could be better maintained in VSTM. Unlike other studies that only made changes to the object (e.g., Hollingworth and Henderson, 2000), we made changes to either object or background context in a trial. Participants had to remember not only the objects but also the backgrounds and retain them in short-term memory for a while before they could compare them to the test scenes. Furthermore, since most visual stimuli can be encoded verbally (Ceraso, 1985), we used an articulatory suppression task to inhibit the verbal recoding of the visual stimuli. In this way, we addressed the visual short-term memory for scenes because participants had to visually remember the whole scene during the task. We predicted that the VSTM would be better for foreground objects than for backgrounds because of the superiority of foreground objects in visual processing (Davenport and Potter, 2004), so participants could better detect the change of foreground objects. With regard to the effect of consistency, we did not have a clear hypothesis because both consistent and inconsistent scenes have their own advantages (familiarity hypothesis vs. novelty hypothesis) and previous studies have shown support for both types of scenes.

The second purpose of this study was to examine the role of semantic or gist change in scene VSTM. To address this issue, change involved either keeping or reversing the consistency of the scene. When a change maintains the consistency (e.g., a car on the road was changed into another car on the same road), the semantics did not alter; when a change did change the consistency (e.g., a car on the road was changed into a boat on the same road), the semantics altered. In this way, we were able to manipulate semantic changes. We predicted that VSTM for scenes would be largely modulated by the change of scene gist. Specifically, the change detection performance would be better for a change that altered the consistency (i.e., a consistent scene changed into an inconsistent scene or an inconsistent scene changed into a consistent scene) compared to a change that did not alter the consistency (i.e., a consistent scene changed into another consistent scene or an inconsistent scene changed into another inconsistent scene).

## Experiment 1

The first experiment investigated how the semantic relationship between foreground objects and background contexts modulated short-term memory for scenes in a one-shot change detection paradigm. We also examined whether this memory could be modulated by semantic or gist change.

### Method

#### Participants

Nineteen right-handed college students (five males, mean age 21.6) participated in this experiment for payment. All participants had reported normal or corrected-to-normal vision. The study followed the tenets of the Declaration of Helsinki, and informed consent was obtained from all participants.

#### Materials

Forty-eight black-and-white scene images were used. Each consisted of a single object in a natural setting (e.g., a car on the road). These images were generated using Adobe Photoshop CS2 software. First, three sets of object and background images were downloaded from the Internet, except for one background image adopted from Davenport and Potter (2004) (see **Table 1**). For each object or background identity (e.g., car and road), two different images were found (e.g., two different car images and two different road images). Then the images were converted into black-and-white by Photoshop. Finally, each object was pasted on all background images in the same set to create scene images (e.g., a car pasted on a road). So the combined scene images could be either consistent (e.g., a car on the road) or inconsistent (e.g., a car on the river). All images were resized to 280 × 210 pixels and presented on a monitor with a gray background at around 57 cm away from participants, the scenes subtended a visual angle of 12.1° horizontally and 9.3° vertically.

**Table 1:** Three image sets used in the study.

	Consistent scenes	Inconsistent scenes
Set 1	Bird – Sky Sea turtle – Underwater	Sea turtle – Sky Bird – Underwater
Set 2	Car – Road Boat – River	Boat – Road Car – River
Set 3	Football player – Football field Herdsman – Grassland	Herdsman – Football field Football player – Grassland


*Each set contains two consistent object-context pairs and two inconsistent object-context pairs. Inconsistent scenes were made by swapping the objects in consistent object-context pairs in each set*.

#### Design and Procedure

**FIGURE 1:**
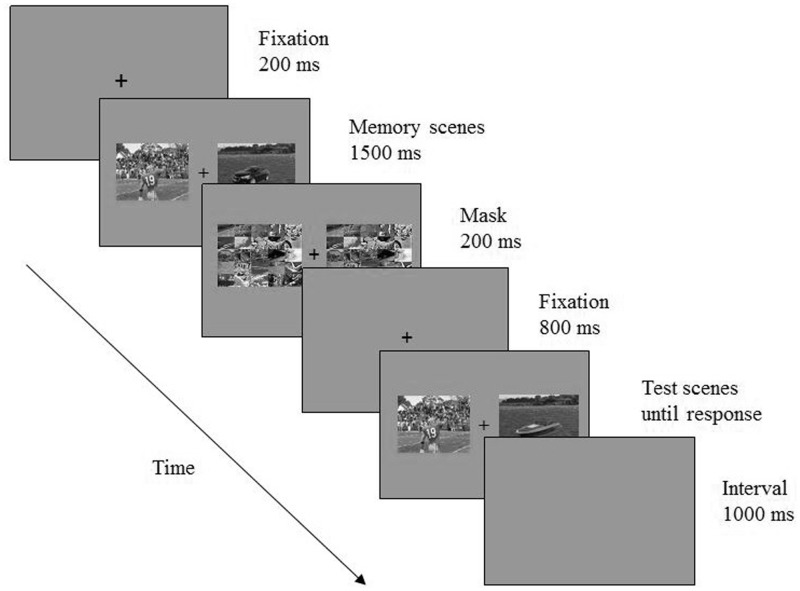
Trial events of Experiment 1. Image courtesy of Davenport and Potter (2004).

A masked change detection paradigm was used. As illustrated in **Figure 1**, each trial began with a fixation point for 200 ms, followed by a memory array for 1500 ms. The memory array consisted of two scene images whose centers were 6.5° to the left and right side of the fixation respectively. Then the two images were replaced by two masks, which appeared for 200 ms. After an 800 ms interval, a test array appeared and participants were instructed to decide whether memory and test arrays were identical or different. The memory and test arrays were identical on half of these trials and different on the other halves. On change trials, the changing part of the scene could be either the foreground object or the background. Participants were asked to left click the mouse when no change was detected or to right click the mouse on the image that were detected as having been changed. Thus participants had to make two judgments consecutively on change trials—to decide whether there was a change and which image had changed—to accomplish the task. A correct choice on change trials required both selections to be correct. Participants were also told to respond as accurately as possible regardless of speed.

Image presentation and behavioral response collections were controlled by E-prime software (Psychology Software Tools, Inc.). Participants sat in a dimly lit room, at about 57 cm from the computer screen (refresh rate: 75 Hz). There were 384 experimental trials in total, with a 1000 ms blank interval between trials. Each participant completed at least 20 practice trials before they proceed to the experimental trials.

To rule out potential verbal encoding (Ceraso, 1985), we used an articulatory suppression task in which participants were instructed to repeat “one-two-three-four” verbally since the presentation of the memory array till response was made on each trial. Articulatory suppression task is an effective method for inhibiting verbal recoding of the visual stimuli (Besner et al., 1981).

### Results and Discussion

When we were computing the detection rate (DR), only judgments on change trials were calculated. On change trials, a correct response means that participants judged correctly in terms of whether there was a change and which image had changed.

**FIGURE 2:**
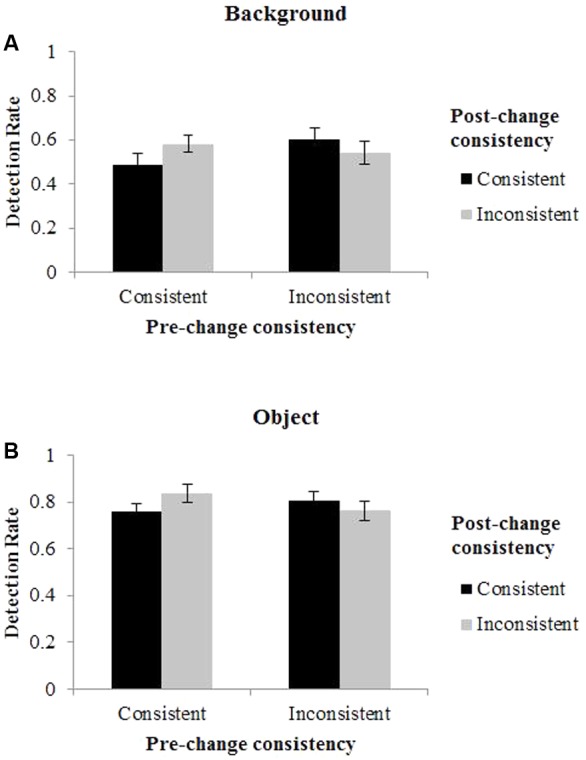
Results of Experiments 1. The columns show detection rates (DR) for **(A)** background, and **(B)** foreground object. Error bars denote standard error of the mean (SEM).

Detection rates are presented in **Figure 2**. A 2 (pre-change consistency: consistent vs. inconsistent) × 2 (post-change consistency: consistent vs. inconsistent) × 2 (changing part: objects vs. background) repeated measure analysis of variance (ANOVA) was conducted on DR. Pre-change consistency referred to the original consistency of memory scenes on change trials and post-change consistency referred to the consistency of the test scenes. The results of the ANOVA showed that the main effect of pre-change consistency was not significant, *F*(1,18) = 1.05, *p* = 0.320, η^2^ = 0.0015, suggesting that the original consistency of the scenes on change did not affect the detection of change. The main effect of changing part was significant, the DR for foreground objects (0.79) was significant higher than that for backgrounds (0.56), *F*(1,18) = 45.84, *p* < 0.001, η^2^ = 0.531. There was a significant interaction between changing part and pre-change consistency, *F*(1,18) = 5.29.60, *p* = 0.034, η^2^ = 0.006. Simple effect analysis showed that the DR was marginally significantly higher for the change of inconsistent scenes than for that of consistent ones when the changing part was background (*p* = 0.058). This difference disappeared when the changing part was foreground object. More importantly, there was also a significant interaction between pre-change consistency and post-change consistency, *F*(1,18) = 11.20, *p* = 0.004, η^2^ = 0.045. Simple effect analysis showed that when post-change scenes were consistent, the DR was higher for inconsistent scenes (0.71) than consistent scenes (0.62); when post-change scenes were inconsistent, the DR was higher for consistent scenes (0.71) than inconsistent scenes (0.65). Together, the results suggested that the DR was higher when consistency changed compared to when consistency did not change. Other interactions were not significant.

**FIGURE 3:**
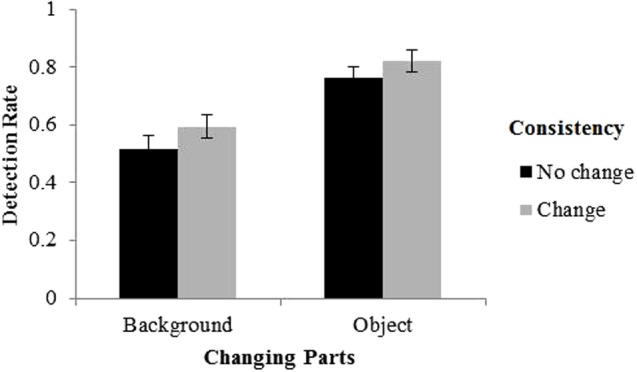
Detection rate as a function of the change of consistency and changing part. Error bars denote standard error of the mean (SEM).

To better visualize the effect of consistency change, we divided the changes into two categories: (1) changes that altered consistency thus changed semantics, which consisted of the changes from a consistent scene to an inconsistent scene or from an inconsistent scene to a consistent scene; and (2) changes that did not alter consistency thus did not change semantics, which consisted of the changes from a consistent scene to another consistent scene or an inconsistent scene to another inconsistent scene. The results were illustrated in **Figure 3**.

Familiarity hypothesis predicted higher detection rates for consistent pre-change scenes, and novelty hypothesis predicted higher detection rates for inconsistent pre-change scenes. To our surprise, neither the familiarity hypothesis nor the novelty hypothesis could predict the results, since consistent scenes seemed to be maintained as well as inconsistent ones in VSTM. The result may suggest that both familiarity and novelty played their roles in the experiment. However, this result is different from previous studies. Possible reasons for the discrepancies are discussed in the General Discussion. Remarkably, participants detected changes that altered scene consistency better than changes that did not alter scene consistency. Since consistency changes signify semantic content (i.e., gist) changes as well, the finding might indicate that people are sensitive to the change of gist, as revealed by Sampanes et al. (2008). Together, with regard to the role of consistency in scene VSTM, the experiment suggests that the change of consistency, rather than the consistency *per se*, is more important.

The experiment also showed that the DR of foreground objects was significantly higher than that of backgrounds, though the areas occupied by backgrounds were larger than that by foreground objects. This result is in line with the findings of Davenport and Potter (2004). They attributed this superiority to the special status of foreground objects in processing—objects may automatically attract attention while the processing of backgrounds may require greater attention resources.

## Experiment 2

The results of Experiment 1 imply that the change of scene consistency plays a more important role than the pre-change consistency in detecting changes in the scene. However, low-level factors may get entangled with the effect of consistency. Although we had attempted to minimize the influence of low-level perceptual features when preparing the experimental stimuli, it was likely that they still played a role in Experiment 1. Therefore, the effect of consistency change in Experiment 1 might actually derive from low-level perceptual features rather than high-level semantic properties of the images.

In Experiment 2, we tried to test whether the differences observed in Experiment 1 was caused by low-level factors. We eliminated the semantic meanings of the scenes using a phase scrambling method. The images after phase scrambling maintained the low-level features (Honey et al., 2008). If the effects discovered in Experiment 1 were caused by low-level physical features, then we should observe the same result pattern as in Experiment 1. Otherwise if the effects were caused by scene consistency, then the pattern should be different in Experiment 2.

### Method

#### Participants

Twenty right-handed college students (eight males, mean age 21.5) participated in the experiment for payment. All participants had reported normal or corrected-to-normal vision, and normal color vision.

#### Materials

**FIGURE 4:**
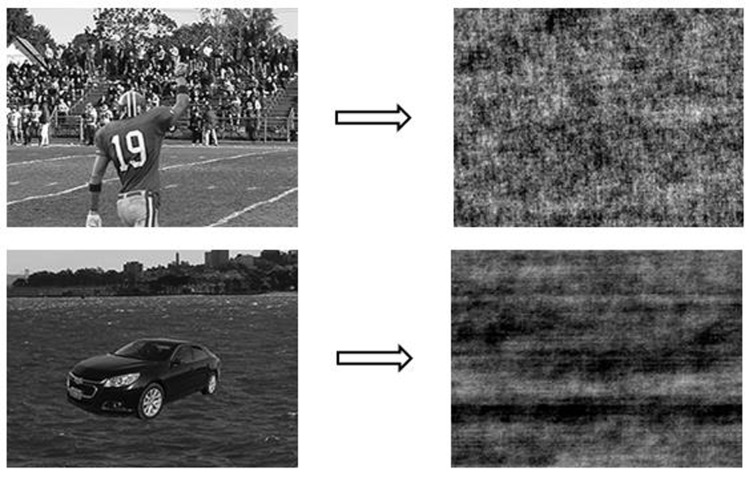
Examples of intact images used in Experiment 1 (the two images on the left: a football player in a football field and a car on a river) and their phase-scrambled version used in Experiment 2 (the two images on the right). Image courtesy of Davenport and Potter (2004).

A phase-scrambled version of images from Experiment 1 was used (**Figure 4**). Phase-scrambling method scrambles the images in the Fourier phase domain and maintains the Fourier amplitude spectrum across orientations and spatial frequencies. This method has been extensively used to create control (i.e., meaningless) stimuli because it eliminates high-level information while basic physical features are well-maintained (Honey et al., 2008). Thus the phase-scrambled images in the present experiment did not contain high-level information including semantic consistency.

#### Design and Procedure

Experimental design and procedure were identical to those in Experiment 1.

### Results and Discussion

**FIGURE 5:**
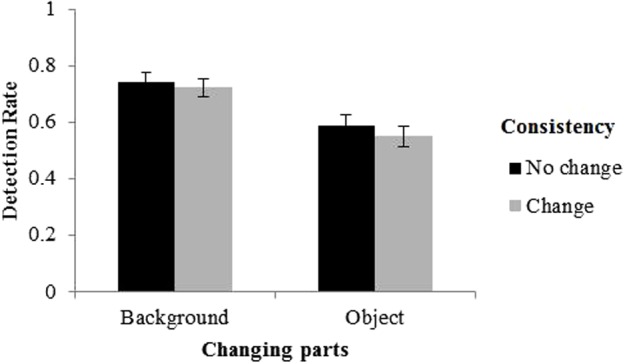
Results of Experiment 2. Error bars denote standard error of the mean (SEM).

Since we were interested in the effect of consistency change, we collapsed the data across the two variables, i.e., “pre-change consistency” and “post-change consistency,” to form a single variable, i.e., “the change of consistency.” The main results are shown in **Figure 5**. A 2 (changing part: objects vs. background) × 2 (the change of consistency: no change vs. change) repeated measure analysis of variance (ANOVA) was conducted on DR. The results did not show a significant main effect of the change of consistency, *F*(1,19) = 3.98, *p* = 0.061, η^2^ = 0.011. This could also be interpreted as a marginally significant effect, but it is worth noting that the DR was higher for changes that did not alter the consistency relative to changes that did alter the consistency, opposite to that in Experiment 1. The results also showed a significant main effect of changing part, with significant higher DR for backgrounds (0.73) than foreground objects (0.57), *F*(1,19) = 72.10, *p* < 0.001, η^2^ = 0.335. The interaction between changing part and the change of consistency was not significant, *F*(1,19) = 0.41, *p* = 0.529, η^2^ = 0.001.

The results of Experiment 2 were obviously different from that of Experiment 1. The effect of consistency change found in Experiment 1 disappeared in Experiment 2, indicating that the effects of low-level changes were roughly similar no matter these changes altered the scene consistency or not. Therefore, we concluded that the effect found in Experiment 1 was indeed due to the change of scene consistency rather than low-level factors. Moreover, when the semantic meanings were removed, the advantage of detecting foreground objects over backgrounds reversed. We speculate that this advantage of background was due to the larger areas occupied by backgrounds than objects, that is, when the meaning of the objects and background were removed, the detection of change depended on the physical areas of the change. Together, Experiment 2 indicates that the advantage of foreground objects observed in Experiment 1 also resulted from the high-level factors of semantic relationship between foreground objects and backgrounds.

## General Discussion

The purpose of this study was to investigate the effect of consistency on short-term memory for scenes. In particular, we aimed to explore whether the change of semantics modulated scene VSTM. In two experiments, we manipulated the semantic consistency and changing part of scenes in a one-shot change detection paradigm. The results suggested a consistency change advantage: changes were more readily detected when scene consistency changed than when consistency remains unchanged. Additionally, there was an obvious foreground object advantage over background.

There are three possible sources of information available when memorizing a visually presented scene: low-level visual information, verbally coded information and high-level semantic knowledge. To prevent the possible verbal coding from VSTM, an articulatory suppression task was used. Therefore, only low-level visual information and high-level semantic information were playing roles in Experiment 1. In Experiment 2, we further excluded the possible confounding effect of low-level visual information, demonstrating that the discrepancies observed in Experiment 1 resulted from high-level semantic information solely.

Previous studies have shown inconsistent results with regard to whether objects in consistent or inconsistent scenes can be better maintained in tasks requiring short-term memory. For instance, Hollingworth and Henderson (2000) supported an inconsistent scenes advantage whereas Spotorno et al. (2013) supported the opposite. In the present study, the changes in consistent and inconsistent scenes were equally detectable, though there was a trend for the inconsistent scene advantage if the change occurred to the background. A potential reason for the different results is due to the varying stimulus durations. Eye movement studies have shown that the very first saccade is rarely directed to the location of the inconsistent object when viewing a scene (Loftus and Mackworth, 1978; De Graef et al., 1992). However, once inconsistent objects are fixated, they are fixated for a longer time compared to consistent objects (e.g., Loftus and Mackworth, 1978). Consistent with this result, Gordon (2004) showed that attention shifted from consistent objects to inconsistent ones as the duration of scenes increased. When the duration was short (42 or 70 ms), attention tended to direct to consistent objects. As the duration increased to approximately 150 ms, more attention was allocated to inconsistent objects. In the study of Spotorno et al. (2013), scenes were presented for only 120 ms, so consistent scene advantage was more likely to occur. By contrast, scenes were presented for a much longer time, 250 and 480 ms respectively, in study of Hollingworth and Henderson (2000) and study of Stirk and Underwood (2007). According to Gordon (2004), inconsistent scene advantage should occur after 150 ms, which was confirmed by the two studies. In our study the scenes were presented for even longer time (1500 ms but two scenes presented simultaneously), so participants had enough time to disengage from inconsistent objects and shift their attention to consistent objects or the background elements. In this sense, the long duration used in the current study should be sufficient enough for a relatively complete representation of the scenes. As a result, the advantage of consistent scenes because of scene schema or the advantage of inconsistent scenes because of attention attracting was canceled out, and no difference exhibited. Therefore, we speculate that the null result was not because neither familiarity nor novelty was playing a role in scene VSTM; conversely, it was because both novelty and familiarity were playing a role when enough time was available for encoding and consolidation.

A key finding of the current study was that participants were more sensitive to the change of consistency, suggesting a top–down semantic modulation to VSTM. Two possible reasons might explain this result. First, consistency may affect the detection of changes in scenes through the detection of gist alteration. The gist of natural scenes can be extracted very rapidly (e.g., Joubert et al., 2007). However, this rapid extraction may only apply to consistent scenes. For an inconsistent scene, it is difficult to form an integrated gist because the meaning of foreground object conflicts with that of the background. As a result, when the consistency changes, so does the gist. The change of gist has crucial influences on subsequent cognitive activities such as scene recognition. It has been shown that a change that alters the gist is easier to detect compared to a change that does not alter the gist (Sampanes et al., 2008). Thus when scene consistency changed in the present study, gist of the scene also changed, leading to a higher DR.

Second, consistency may mediate change detection of natural scenes through semantic informativeness. Semantic informativeness is defined as how much information an object carries or provides to the scene (Hollingworth and Henderson, 2000). A semantically inconsistent object (e.g., a bird flies underwater) provides information that is not carried by other elements in the scene, so it is more informative; a semantically consistent object (e.g., a fish swims underwater) is less informative in the sense that it provides information that overlaps with other elements in the scene. This could be understood from a semantic network perspective: for the scene “a fish swims underwater” and the scene “a bird flies underwater,” the node representing river is connected with the node of fish but not bird in the semantic network, so information carried by river overlaps that carried by fish rather than that by bird. Hollingworth and Henderson showed that the change detection latency was shorter for more informative inconsistent objects than less informative consistent objects. Support also comes from Spotorno et al. (2013) who showed that changes to “diagnostic” objects (i.e., objects that were unique, distinctive to and aided the understanding of a scene) were easier to detect. For example, a toaster usually appears in the kitchen whereas a mobile phone can appear in many place (tables, sofas, beds, pockets, and bags, etc.), so a toaster is a more diagnostic object than a mobile phone when considering scene consistency. In essence, diagnostic objects are informative objects, so this study also suggests that people are sensitive to informativeness change. Therefore, informativeness changes if a change alters consistency in the present study, giving rise to a higher DR.

The present study also showed a foreground object advantage over background in change detection. This finding is in line with gestalt principles of figure/ground perception and studies showing an advantage of objects over backgrounds in attracting attention (e.g., Cole and Hughes, 1990) and naming (Davenport and Potter, 2004). We also demonstrated that this advantage resulted from high-level semantic factors rather than from low-level factors because DRs were the same for background and foreground object when semantic meanings of images were removed. A compelling interpretation is that foreground object possesses a special status in visual processing—it may automatically attract attention (Davenport and Potter, 2004). Therefore, foreground object gains more cognitive resources, resulting in a better performance in memory task.

## Conclusion

The current study adopted a one-shot change detection paradigm to examine the role of semantics change in scene VSTM. The results revealed that changes were more accurately detected when scene consistency changed than when consistency did not change. This advantage was not due to the low-level physical change, but was due to the high-level semantic consistency between foreground object and background. Together, the study suggests that the change of semantic content modulates VSTM.

## Ethics Statement

This study was carried out in accordance with the recommendations of Ethics Committee of Human Experimentation at the Institute of Psychology with written informed consent from all subjects. All subjects gave written informed consent in accordance with the Declaration of Helsinki. The protocol was approved by the Ethics Committee of Human Experimentation at the Institute of Psychology.

## Author Contributions

MG participated in all the stages of the study, i.e., study design, data collection, data analysis and manuscript writing. YX participated in study design, data interpretation, and manuscript revision as well as acted as corresponding author. XX participated in data collection and manuscript revision. XF supervised development of work and helped with manuscript revision.

## Conflict of Interest Statement

The authors declare that the research was conducted in the absence of any commercial or financial relationships that could be construed as a potential conflict of interest.
